# Distinct Brain and Behavioral Benefits from Cognitive vs. Physical Training: A Randomized Trial in Aging Adults

**DOI:** 10.3389/fnhum.2016.00338

**Published:** 2016-07-18

**Authors:** Sandra B. Chapman, Sina Aslan, Jeffrey S. Spence, Molly W. Keebler, Laura F. DeFina, Nyaz Didehbani, Alison M. Perez, Hanzhang Lu, Mark D'Esposito

**Affiliations:** ^1^Center for BrainHealth®, The University of Texas at DallasDallas, TX, USA; ^2^Advance MRI, LLCFrisco, TX, USA; ^3^The Cooper InstituteDallas, TX, USA; ^4^Department of Psychiatry, University of Texas Southwestern Medical CenterDallas, TX, USA; ^5^Department of Radiology and Radiological Science, Johns Hopkins UniversityBaltimore, MD, USA; ^6^Helen Wills Neuroscience Institute, University of CaliforniaBerkeley, CA, USA

**Keywords:** aging, cognitive training, aerobic exercise, CBF, executive function, memory, ClinicalTrials.gov, NCT# 00977418

## Abstract

Insidious declines in normal aging are well-established. Emerging evidence suggests that non-pharmacological interventions, specifically cognitive and physical training, may counter diminishing age-related cognitive and brain functions. This randomized trial compared effects of two training protocols: cognitive training (CT) vs. physical training (PT) on cognition and brain function in adults 56–75 years. Sedentary participants (*N* = 36) were randomized to either CT or PT group for 3 h/week over 12 weeks. They were assessed at baseline-, mid-, and post-training using neurocognitive, MRI, and physiological measures. The CT group improved on executive function whereas PT group's memory was enhanced. Uniquely deploying cerebral blood flow (CBF) and cerebral vascular reactivity (CVR) MRI, the CT cohort showed increased CBF within the prefrontal and middle/posterior cingulate cortex (PCC) without change to CVR compared to PT group. Improvements in complex abstraction were positively associated with increased resting CBF in dorsal anterior cingulate cortex (dACC). Exercisers with higher CBF in hippocampi bilaterally showed better immediate memory. The preliminary evidence indicates that increased cognitive and physical activity improves brain health in distinct ways. Reasoning training enhanced frontal networks shown to be integral to top-down cognitive control and brain resilience. Evidence of increased resting CBF without changes to CVR implicates increased neural health rather than improved vascular response. Exercise did not improve cerebrovascular response, although CBF increased in hippocampi of those with memory gains. Distinct benefits incentivize testing effectiveness of combined protocols to strengthen brain health.

## Introduction

Life expectancy has increased steadily over the past century; but the downside of longer life is fear of losing the mental capability to sustain independent living. Adults are more worried about cognitive health than cancer or heart health for the first time ever (IOM Report, 2015). Whereas, many fear Alzheimer's disease, an estimated 87% of adults will not be diagnosed with Alzheimer's disease (Wagster et al., 2012). Nonetheless, many normally aging adults without dementia manifest continuous and significant cognitive losses. These cognitive losses are most frequently identified in domains of executive function/cognitive control and memory (Cepeda et al., 2001; Mahncke et al., 2006a; Levine et al., 2007). Executive function refers to the ability to flexibly select and inhibit information; to maintain, update, and abstract meanings; and to fluidly innovate ideas, while at the same time maintaining an active goal in mind (Levine et al., 2007; Badre, 2008; Vas et al., 2015). Executive functions support real life activities that allow humans to orchestrate the complexities of daily tasks and responsibilities such as reasoning, problem-solving, flexible thinking, generalizing rules, planning, and decision-making, among others (Anguera et al., 2013). With regard to memory function, older adults consistently perform lower than younger individuals and the gap widens with advancing age (Salthouse, 1994).

In parallel with cognitive control and memory losses, a growing body of work identifies degradation of structural and functional aspects of brain systems with age (Raz et al., 1997; Kennedy and Raz, 2009; Lu et al., 2011). Age-related brain changes are prominently found in prefrontal and temporal/hippocampal brain regions. The prefrontal cortex (PFC) and its complex networks play a pivotal role in higher-order executive functions (D'Esposito and Chen, 2006; Badre, 2008). The hippocampal regions, part of the medial temporal lobes, have been shown to support memory (Rodrigue et al., 2013). Taken together, evidence reveals that the normally aging brain may represent a developmental model of failing frontal networks, diminishing executive function systems, and declining memory (D'Esposito and Chen, 2006). Fortunately, the insidious cognitive decline documented in normal aging appears to be modifiable and even reversible to some extent (Greenwood and Parasuraman, 2010). The aging brain's retained capacity to show dynamic functional and beneficial changes in response to training is being demonstrated through employing emerging brain imaging methodologies and analytics (Colcombe et al., 2004; D'Esposito and Chen, 2006; Greenwood and Parasuraman, 2010; IOM Report, 2015). Thus, integrating outcome data from both brain and cognitive measures will help elucidate the efficacy of non-pharmacological interventions such as cognitive and physical training protocols (Erickson et al., 2011; Takeuchi et al., 2013; Chapman et al., 2015).

The potential benefits of a wide array of cognitive training and exercise protocols are being tested. Cognitive training protocols that entail strategy-use, reasoning, or multi-domain approaches have shown to induce broad-based, generalized, and more lasting gains in older healthy adults than achieved by training of specific cognitive processes, such as memory (Ball and Birge, 2002; Chapman and Mudar, 2014; McDaniel et al., 2014; Strenziok et al., 2014). Evidence for age-related benefits from cognitive training protocols have been identified from short-term advanced reasoning training as reflected in the SMART protocol (Anand et al., 2011; Chapman and Mudar, 2014; Chapman et al., 2015), non-verbal reasoning arm of the ACTIVE trial (Rebok et al., 2014), strategic goal setting in Goal Management Training (Levine et al., 2007; Karbach and Kray, 2009), multi-domain computer training (Shatil, 2013), and Posit Science's computer training program (Mahncke et al., 2006a,b; Strenziok et al., 2014). Strategy-based cognitive training protocols, such as SMART (Chapman et al., 2015) and Goal Management Training (Levine et al., 2000; D'Esposito and Chen, 2006), have shown to induce broader transfer of benefits on measures such as abstraction, working memory, verbal reasoning, and inhibition, as well as reducing depressive symptoms and improving real life function compared to training of specific cognitive processes (Anand et al., 2011; Barnes et al., 2013; Chapman and Mudar, 2014; Vas et al., 2015). With regard to exercise regimens, the evidence supports the intuitive view that any exercise program is better than none (IOM Report, 2015). Nonetheless, considerable evidence suggests that programs involving aerobic exercise (Kramer et al., 1999; Colcombe et al., 2004; Chapman et al., 2013) as compared with non-aerobic exercise such as stretching and toning (Aberg et al., 2009) may be associated with greater benefits especially observed to improve executive and memory functions.

A handful of studies have examined the cognitive benefits of physical vs. cognitive training (Oswald et al., 2006; Shatil, 2013). In a carefully controlled, four-condition randomized trial in healthy older adults ranging in age from 65 to 93 years, Shatil and colleagues reported findings implicating a selective benefit of a multi-domain cognitive training program called CogniFit (Shatil, 2013) as compared to physical training (mild aerobic training) and an active control group (discussing a specific book) delivered in 32 h over a 4-month period. Cognitive training alone and combined cognitive/physical training improved performance on a broad array of cognitive measures in healthy older adults. In contrast, no significant gains were observed on any of the cognitive tasks in the mild aerobic training group alone or the active control group (Shatil, 2013). Their findings suggested that enhancement of mental performance necessitated cognitive training as a pivotal element to achieve gains since no significant cognitive gains emanated from aerobic training alone. Otherwise, they offered that cognitive gains from physical exercise may only be realizable after longer exercise training intervals, such as 1 year of training. In another study of normally aging adults 75–93 years, only combined cognitive (i.e., speed of information processing, memory, storage, retrieval, attention) and physical training (i.e., targeting balance, flexibility, and motor coordination) showed cognitive benefits (Oswald et al., 2006). The combined training had more lasting benefits than separate physical or cognitive training alone. The proposed interpretation was that the physical training may have improved the metabolic activity of the brain, but the gain was only realized by enrichment of cognitive training that exploited the neural demand through challenging cognitive tasks. Therefore, there remains a gap in our basic understanding of the unique contributions of each training to cognition and brain function. In this study, we aimed to elucidate distinct benefits of each training before exploring the benefits of combined trainings.

Both the Shatil and Oswald studies provided key insights into benefits of cognitive and physical exercise training with results that could be further informed by subsequent inclusion of brain function measures such as cerebral blood flow (CBF) and cerebral vascular reactivity (CVR). Emerging evidence suggests that functional brain changes measured by CBF and CVR occur more rapidly and frequently than structural changes (Bruel-Jungerman et al., 2007). CBF is an important physiological parameter that is responsible for energy homeostasis of the brain. Due to neuronal tissue's high metabolic demand, a tight regulation between CBF and neuronal activity is required (Raichle and Gusnard, 2002). Under normal physiological conditions, the total brain blood flow is constant, partly due to large feeding arteries. However, in case of high metabolic demand, the amount of blood flow is increased with vasodilation of blood vessels (Leithner and Royl, 2014). The ability of the blood vessels to vasodilate is called CVR and is an important marker for brain vascular function and reserve. One way to induce a CVR response is by exposing the brain to CO_2_ (Yezhuvath et al., 2009). Previous research suggests that global CBF and CVR decline with increasing age and the changes most notably affect the frontal lobes (Lu et al., 2011). Combining CBF with CVR parameters provides a way to assess changes in different aspects of brain health, i.e., improvements related to either vascular and/or neuronal health.

Researchers have started exploring functional brain changes in response to shorter interventions such as aerobic exercise and cognitive training. Smith and colleagues found that global CBF increased by 20% following exercise as well as motor cortex CBF during a finger tapping experiment (Smith et al., 2010). MacIntosh and colleagues assessed the resting brain perfusion at 10 and 40 min after a single bout of exercise and found that the resting CBF of bilateral hippocampi decreased at both intervals (MacIntosh et al., 2012). Additionally, we previously showed that the resting CBF of bilateral anterior cingulate cortex increased after 12 weeks of exercise training compared to a wait-list control group (Chapman et al., 2013). In terms of cognitive training programs, a 4 week working memory training was shown to increase the resting regional CBF of the right lateral PFC (Takeuchi et al., 2013). In our previous work, we found that changes to resting CBF preceded changes to structural white matter changes (Chapman et al., 2015). Despite these positive reports, the existing evidence does not allow us to glean a direct comparison of neural benefits from cognitive vs. physical training in a comparable group employing CBF and CVR measures. The ability to measure CVR relatively safely has only recently been developed; therefore, no known study has investigated CVR changes in response to cognitive and aerobic interventions.

The present study directly compared the effects of cognitive vs. physical training on cognitive performance and the resting state CBF and CVR measurements in a relatively short period of time, i.e., 12 weeks. In our previous work, we found global CBF change to be an informative marker of improved cognitive brain health in normally aging adults in response to advanced reasoning training when compared to a control group (Chapman et al., 2015). In a comparison study of aerobic exercise vs. control group study, we found regional blood flow changes but not a global change. The current study expands on this prior foundational work by incorporating vascular and perfusion based MR techniques to help elucidate the distinct impact of cognitive vs. physical training on the brain and behavior.

Thus, this study explored a direct comparison of two non-pharmacological training protocols (i.e. cognitive training vs. physical training) by showing their effects on behavior and brain. Specifically, we explored the potential to enhance executive function and memory in normally aging adults and investigated their impact on brain function using resting state CBF and CVR measures. We postulated that the complex reasoning training would increase global CBF due to the known tight coupling of the neurovascular system induced by cognitive stimulation. In contrast, aerobic training was predicted to improve vascular health in the brain as measured by the brain's vascular response to CO_2_. The aim was to advance our understanding of the distinct contribution of cognitive vs. exercise stimulation to neural, cerebrovascular, and cognitive health.

## Materials and methods

### Participants

The study was approved by the ethical review board of the Texas Southwestern Medical Center at Dallas, University of Texas at Dallas and The Cooper Institute. All participants provided written informed consent before participating in the study. Participants (*n* = 137) were recruited from a database at The Cooper Institute, through flyers/advertisements placed in local newspapers and magazines, and our institutions' websites. Participants were included if their self-reported physical activity was described as participating in aerobic physical activity less than or equal to twice a week for 20 min for at least the preceding 3 months. This history was ascertained in the initial phone screen by The Cooper Institute and confirmed during the phone screen by the Center for BrainHealth, given the unreliable nature of self-reported physical activity. All levels of physical activity below this cut-point were considered for enrollment including those who were physically inactive.

All participants underwent the Telephone Interview of Cognitive Status-Modified (TICS-M) to screen for dementia (de Jager et al., 2003), the Montreal Cognitive Assessment (MoCA) to screen for cognitive impairment (Nasreddine et al., 2005), the Beck Depression Inventory 2nd edition (BDI-II) to screen for depressive symptoms (Beck and Brown, 1996), and complete medical, physical, and laboratory assessments by a physician to ensure good general health. The criteria for inclusion were no history of neurological or psychiatric conditions, normal IQ range, native English speakers, and minimum of high school diploma. Exclusionary criteria included: MR scanning contraindications, cognitive status (TICS-M < 28 and MoCA < 26), depression indication (BDI > 14), left-handedness, body mass (BMI > 40, $\frac{\text{mass}\left(\text{kg}\right)}{\text{height}{\left(\text{m}\right)}^{2}}$). The participant's BMI was limited to 40 to ensure participants' bodies and heads fit in the scanner and the head coil.

After passing the initial phone screens, individuals were further evaluated at The Cooper Institute for any medical history including medications and physical activity history and a physical examination including blood pressure, height/weight, BMI, and waist circumference. At this point, the participants underwent graded exercise stress testing to evaluate for obstructive coronary artery disease or other findings that would represent contraindications to exercise and for measurement of the cardiorespiratory fitness level (VO_2_ Max). VO_2_ Max is a measure of maximum capacity of the body to use and transport oxygen. Participants were tested utilizing a Lode—Excalibur Sport cycle ergometer, (Groningen, Netherlands) and a Jaeger Oxycon Pro, (Hoechberg, Germany), to assess VO_2_ Max and exercise electrocardiogram. Maximum effort was strongly encouraged during the testing and all included participants had reached at least 85% of their maximum predicted heart rate to ensure that evaluation for ischemic findings was clinically acceptable. Blood pressure and ECG responses to exercise were monitored and ratings of perceived exertion (RPE) were obtained using the Borg scale: 6 (no exertion) to 20 (extremely difficult). Further exclusion criteria included abnormal electrocardiographic response, significant hypertensive blood pressure response to exercise, inability to reach 85% of maximum predicted heart rate for age or not completing maximal exercise effort/test.

Participants who met the above inclusion criteria (*n* = 67) were randomized using a block randomization schedule stratified by gender (ClinicalTrials.gov, NCT# 00977418) into one of three groups: cognitive training (*n* = 25), physical training (*n* = 21), or wait-listed control (*n* = 21) group. However, seven participants in cognitive training, three participants in physical training and two participants in control groups declined to participate in the study. Thus, the *final participant numbers* were: cognitive training (CT, *n* = 18), physical training (PT, *n* = 18), and control (CN, *n* = 19), see Figure S1. The present study included 36 cognitively normal adults (mean age = 63.5 ± 3.8; 56–75 years of age) who completed the cognitive or physical training programs. Participants were not compensated for their involvement in this study. Written informed consent was obtained from all subjects in accordance with the Institutional Review Board (IRB) of our academic institutions: The University of Texas at Dallas, The University of Texas Southwestern Medical Center and The Cooper Institute.

### Training protocols

Both the Cognitive and Physical training programs were administered three times per week over 12 weeks at the Center for BrainHealth and The Cooper Institute, respectively. Participants were required to attend at least 90% of the training, missing no more than 3 of the 36 sessions. The trainers for both arms were unaware of study hypotheses but encouraged the participants to exercise and apply the cognitive training strategies outside of the training sessions.

### Cognitive training program

The cognitive training was led by a clinician who had previously completed a three-stage training process prior to study implementation consisting of reading about the program, observing a trained clinician, and leading training sessions under supervision to insure treatment fidelity. Cognitive training occurred 1 h per week in small groups (*n* ≤ 5) with two 1-h unsupervised individual work sessions where individuals completed pencil and paper homework assignments on their own at home (i.e., supervised training = 12 h, individual unsupervised work = 24 h). During these “solo” sessions, individuals were given assignments to practice the core strategies as described in the next section. Participants' independent work was reviewed by the trainer prior to the next group training session and discussed at group meetings. No separate contact was held between sessions. Record logs of time and assignment completion were kept for the individual work.

The cognitive training group underwent an evidenced-based, manualized cognitive training program referred to as Strategic Memory Advanced Reasoning Training or SMART (Chapman and Mudar, 2014; Chapman et al., 2015). The SMART program trains metacognitive strategies to improve top-down thinking using three core cognitive control functions of strategic attention, integrative reasoning, and innovation. The training is strategy-based rather than content specific and provides practical ways to employ the strategies in real life contexts. *Strategic Attention* focuses on strategies to prepare the brain to efficiently manage time and cognitive resources by prioritizing goal setting to the top two tasks each day, intentionally single-tasking, blocking distractions and inhibiting irrelevant input, and building in regular mental breaks. *Integrative Reasoning* teaches individuals to synthesize information at deeper levels of interpretation by abstracting the essence or extracting key goals for tasks. Strategies to build Integrative Reasoning exert cognitive control to “zoom in” to the details or steps to a goal, then rapidly “zoom out” to synthesize, and abstract big picture ideas/goals, followed by “zooming deep and wide” to construct generalized application of derived ideas, interpretations, or goals-completed. The top-down processes facilitated by *Innovative Strategies* encourage fluid and flexible thinking, perspective-taking and problem solving. SMART trains individuals to (1) approach a task with a brain-set ready to think deeply, (2) continually synthesize meanings from data encountered in everyday life (e.g., medical information, investment information, movies, lectures, newspaper articles, travel highlights), and (3) practice innovative thinking by generating diverse interpretations, perspectives, and solutions to daily work or leisure activities. For example, imagine listening to your doctor's advice about a new condition you have just been diagnosed where the possible choices are overwhelming. Individuals are trained to (a) strategically inhibit taking in excessive, often poorly vetted information from the web, focusing on their top two concerns; then (b) use integrative reasoning by “zooming in” to the most concerning aspects of the disease, then, “zooming out” to abstract life and health priorities, and next “zooming deep and wide” to consider life impact by acting on or rejecting doctor's recommendations, and finally (c) employ innovation strategy to evaluate pros and cons of treatment options and engage family and trusted friends advice to weigh different perspectives or options. Another brief example is after seeing a movie, discuss it with friends where instead of downloading the entire story (strategic attention); abstract key themes and take home messages applied to your own life context (integrated reasoning) and generate diverse perspectives representing a plethora of possibilities (innovation). Participants were encouraged to incorporate these three core cognitive processes as often as possible within the context of their own life activities and goals (Chapman and Mudar, 2014).

### Physical training program

The physical training program was designed in an effort to ensure the participants met the 2008 physical activity guidelines of 150 min per week, as recommended for all adults (http://health.gov/paguidelines/guidelines/chapter4.aspx). The exercise prescription was developed based on the study intervention and the heart rate obtained at VO_2_ max of 50–75%. Three physical exercise sessions of 60 min were done per week. Each session involved 5 min of warm up, 50 min of aerobic exercise, and 5 min of cool down. The training regimen alternated weekly between working out 2 days on an exercise bike, one on a treadmill and vice versa. The warm up consisted of walking on treadmill at a speed of 2–2.4 mph or cycling at 43 Watts for 5 min. The workout consisted of walking on treadmill or stationary cycling for 50 min at a speed determined to be 50–75% of VO_2_ Max. The cool down consisted of walking on treadmill at a speed of 2–2.4 mph or cycling at 43 Watts for 5 min. Every physical training session occurred individually under the supervision of trained personnel with experience in controlled exercise training trials to ensure adherence and safety. Natural occurring social interactions occurred between the trainer and the participant during the training. The participants were monitored at 5 min intervals throughout their exercise session to ensure that they attained and maintained at least 50% of the maximum heart rate seen with VO_2_ max. In addition to heart rate, RPE was assessed every 5 min. As the participants were otherwise healthy and functional, they were encouraged to complete the exercise sessions and work toward increasing intensity throughout the study.

All participants were able to adhere to the exercise prescription since they were initially screened to complete 1 h of physical training without breaks. Participants were encouraged to exercise as frequently as possible outside of the training sessions so they could begin to increase physical activity in real life. A physician was also present onsite in case of emergencies.

### Neurocognitive measures

A battery of neurocognitive measures was administered on a non-training day at three time periods, i.e., baseline/pre-training (T1), mid-training week 6 (T2), and at end of training, week 12 (T3) for cognitive and physical training groups. Cognitive assessment measures were completed in individual, face-to-face testing sessions. All test batteries were pencil and paper measures administered by trained individuals who were masked to training group membership. Furthermore, participants were instructed not to share any information about their group membership to their examiners.

Assessment of near transfer effects of training included three randomized versions of the Test of Strategic Learning (TOSL) to measure the ability to synthesize abstract meanings from complex input. Measurements of far transfer effects included tests of executive function, memory, and complex attention. Measures of executive function included: Wechsler Adult Intelligence Scale 3rd edition (WAIS-III) Similarities (concept abstraction; Wechsler, 1997), Daneman Carpenter (working memory; Daneman and Carpenter, 1980), Delis-Kaplan Executive Function System (DKEFS) Sorting Test (Delis et al., 2001), Controlled Oral Word Association Test (COWAT) category total (semantic verbal fluency) and letter total (phonemic verbal fluency; Lezak et al., 2004), and Trails B (switching; Tombaugh, 2004). Immediate memory was assessed with trial one of the California Verbal Learning Test 2nd edition (CVLT II; two versions used alternatively over time intervals), and immediate and delayed memory was assessed with the Wechsler Memory Scale 2nd edition (WMS-II) Logical Memory subtest. The results are one-tailed due to anticipated improvements in the neurocognitive and physiological measures of each training group.

### Physiological measures

Physiological markers were assessed and documented at The Cooper Institute at three time points on a non-training day: baseline/pre-training (T1), mid-training, week 6 (T2), and upon completion of the training, week 12 (T3). The physiological parameters included: weight (kg), heart rate (beats per min or bpm), VO_2_ Max (ml/kg/min), and RPE scale. The RPE was assessed based on Borg's Scale: 6 (no exertion) to 20 (extremely difficult). The RPE was included to serve two purposes in our study: to get a subject exertion measure from participants and to regulate their exercise activity (increasing the heart rate from 50 to 75%).

### MRI experiment

MRI investigations were performed on a 3 Tesla MR system (Philips Medical System, Best, Netherlands). A body coil was used for radiofrequency (RF) transmission and an 8-channel head coil with parallel imaging capability was used for signal reception. The MRI scans of participants were performed at rest with their eyes open and on a non-training day. We used pseudo-continuous arterial spin labeling (pCASL) sequence to measure CBF at rest (Aslan et al., 2010). CVR measurements were carried out using a BOLD MR sequence. The subjects alternated breathing room air and 5% CO_2_ for 1 min each for a total of 7 min (Yezhuvath et al., 2009). Additionally, a high resolution T1 weighted image was acquired as an anatomical reference. The details of imaging parameters and their processing techniques are provided below.

Imaging parameters for pCASL experiments were: single-shot gradient-echo EPI, field-of-view (FOV) = 240 × 240, matrix = 80 × 80, voxel size = 3 × 3 mm^2^, 27 slices acquired in ascending order, slice thickness = 5 mm, no gap between slices, labeling duration = 1650 ms, time interval between consecutive slice acquisitions = 35.5 ms, TR/TE = 4020/14 ms, SENSE factor 2.5, number of controls/labels = 30 pairs, RF duration = 0.5 ms, pause between RF pulses = 0.5 ms, labeling pulse flip angle = 18°, bandwidth = 2.7 kHz, echo train length = 35, and scan duration 4.5 min. The hypercapnia BOLD imaging parameters were: single shot gradient echo EPI sequence, TR/TE/flip = 2000 ms/25 ms/80°, 43 axial slices, slice thickness = 3.5 mm, FOV = 220 × 220 mm^2^, matrix size = 64 × 64, and scan duration = 7 min. Hypercapnia was administered using a Douglas bag with a two-way valve to switch between 1 min blocks of 5% CO_2_-breathing (mixed with 21% O_2_ and 74% N_2_) and air-breathing. Physiologic parameters, including end-tidal (Et) CO_2_, breathing rate, heart rate, and arterial oxygenation (sO_2_), were recorded during the scan (MEDRAD, Pittsburgh, PA and Novametrix Medical Systems, Wallingford, CT). The high resolution T1 weighted image parameters were: Magnetization Prepared Rapid Acquisition of Gradient Echo (MPRAGE) sequence, TR/TE = 8.3/3.8 ms, shot interval = 2100 ms, inversion time = 1100 ms, flip angle = 12°, 160 sagittal slices, voxel size = 1 × 1 × 1 mm^3^, FOV = 256 × 256 × 160 mm^3^, and duration 4 min.

The pCASL MRI data underwent routine processing (Aslan et al., 2010). PCASL image series were realigned to the first volume for motion correction (SPM5's realign function, University College London, UK). All datasets were within the applied motion threshold of 3 mm translation and 3° rotation. An in-house MATLAB (Mathworks, Natick, MA) program was used to calculate the difference between averaged control and label images. Then, the difference image was corrected for imaging slice delay time to yield CBF-weight image, which was normalized to the Brain template from Montreal Neurological Institute (MNI). This procedure was carried out using a non-linear elastic registration algorithm, Hierarchical Attribute Matching Mechanism for Elastic Registration (HAMMER, University of Pennsylvania, PA). The HAMMER algorithm detects and corrects for region-specific brain atrophy which is commonly seen in elderly subjects. Last, the absolute CBF was estimated in the units of mL blood/min/100 g of brain tissue (Aslan et al., 2010). The absolute whole brain blood flow values were calculated by averaging all the voxels in the absolute CBF (aCBF) map. In the ROI analysis, the hippocampal and parahippocampal regions were defined and combined by automated anatomical labeling (AAL) parcellation template in SPM (Tzourio-Mazoyer et al., 2002). In voxel based analyses (VBA), the individual aCBF maps were spatially smoothed [with full-width half-maximum (FWHM) of 4 mm] to account for small differences in sulci/gyri location across subjects. For subject-level CVR, we followed protocols established previously (Yezhuvath et al., 2012). Briefly, we used EtCO_2_ trace (synchronized with the MRI acquisitions) as a regressor on the BOLD time series to obtain CVR, measured as % BOLD signal change/mmHg EtCO_2_ change. Similar to the CBF maps, HAMMER software was used to normalize the CVR maps to MNI template space for the group level analysis.

### Statistical analysis

At the group level, a general statistical linear model was applied to assess the contribution of cognitive and physical training on neurocognitive measures and CBF. The model included sessions (T1, T2, and T3), group status (cognitive training and physical training) and the interaction between these factors. Two variance components—one due to variability across subjects, and one due to variability in the same subject over time—were included to account for the different levels of variability and estimated by residual maximum likelihood (REML). We were primarily interested in how the groups differed across the training sessions. We hypothesized that the cognitive training group would show an increase in mean of behavioral measures (except “Trails B”; since it is a timed measure and lower score represents faster performance), either by T2 or T3, relative to the physical training group. On the other hand, we hypothesized that the physical training group would show an increase in VO_2_ Max and a decrease in RPE measurement since individuals may perceive less effort in physical training as they adjust to exercise regimen. This hypothesis led to two orthogonal polynomial interaction contrasts: linear and quadratic. The linear interaction contrast tested whether the mean change between the groups increased monotonically from T1 to T3, and the quadratic interaction contrast tested whether the mean change between groups increased maximally at T2 before decreasing (either back to baseline or only partially) at T3.

To control family-wise error (FWE) for cluster extent inference, we used a program based on AlphaSim, called 3dClustsim in AFNI (NIMH Scientific and Statistical Computing Core, Bethesda, MD, USA), which yields the minimum cluster sizes for a pre-specified FWE alpha level, a pre-specified voxel threshold, and an estimated smoothness of the statistic map. This procedure controls false positive activation clusters over the set of all activation clusters throughout the whole brain volume. Therefore, we refer to this inference in Results as FWE corrected. We estimated the smoothness to be 9.9 and 8.1 mm FWHM (inherent smoothness and applied 4 mm kernel smoothing) for the main effect analysis and covariate analysis, respectively. In both cases, we set the cluster-defining threshold to *p* = 0.001. These parameters yielded a minimum cluster size of 154 voxels (1232 mm^3^) and 94 voxels (752 mm^3^), respectively, for a FWE-corrected alpha level of 0.01. Additionally, we evaluated a priori regions of interest, left/right hippocampal CBF, since it has been shown that the hippocampus volume increases with aerobic exercise (Erickson et al., 2011). Significant clusters from the VBA, as well as a priori regions of interest, left/right hippocampus, were further examined for potential relationships with neurocognitive measures, cardiovascular measures (VO_2_ max) and RPE. These relationships were assessed in a GLM framework, similarly as above, with group status a classification variable, linear/quadratic contrasts of NP/VO_2_ Max/RPE as a quantitative explanatory variable and linear/quadratic contrasts of rCBF estimates as dependent variables. Our primary interest was to determine whether these relationships across NP/VO_2_ Max/RPE and rCBF differed between the physical and cognitive training groups (i.e., the interaction term, which allows separate relationships by group). We hypothesized that the physical training group would demonstrate positive associations between physical fitness (improved VO_2_ max) and hippocampus blood flow. As in the previous GLM, the regression coefficients as well as group differences between them were scaled to t-statistics with inference, similarly, based on Student's *t*-distribution. Finally, we applied FDR control at level 10% to minimize false positives from simultaneous inference on neurocognitive and physiological tests.

## Results

### Subject characteristics

The current study uniquely focused on the differential cognitive and neural benefits from either cognitive reasoning or aerobic exercise training. All cognitive training (CT, *n* = 18) and physical training (PT, *n* = 18) participants completed the neurocognitive assessments at each time point. However, several participants in the CT and PT groups were not included in the analysis due to incomplete MRI time points, gross movement of >3 mm, and >3°, artifact or hypercapnia issues (see Figure S1). The final MRI data analyses were conducted on the majority of the participants, shown in Table 1. All PT and CT participants were required to complete at least 90% of training sessions over the 3 month training period which means they completed 32 h or more of the 36 h of training. No participant was excluded due to missing too many sessions to meet the 90% criterion. No significant differences in age, gender, IQ, MoCA, TICS-M were noted between groups (*p* > 0.05). Three participants in the PT group reported adverse events in the study (mild change in EKG, leg cramp, change in blood pressure) which may or may not have been related to the training.

**Table 1:** **Subject characteristics and total number of subjects per group, assessments and MRI technique (mean ± *S.D.*)**.

	**PT (*n* = 18)**	**CT (*n* = 18)**	***p*-values**
Age	64.0 ± 4.3	61.8 ± 3.3	0.20
Gender (M/F)	5/13	8/10	0.31
IQ	117.5 ± 9.9	121.6 ± 8.0	0.18
MoCA	27.8 ± 1.5	27.9 ± 1.4	0.82
TICS-M	30.7 ± 2.0	29.4 ± 2.2	0.08
VO2 Max	19.3 ± 3.3	20.7 ± 5.0	0.29
Participants (*n*)			
Cognitive exams	18	18	
Physical exams	18	18	
pCASL MRI	18	13	
HC BOLD MRI	14	12	

PT, Physical Training; CT, Cognitive Training; IQ, Intelligence Quotient; MoCA, Montreal Cognitive Assessment; TICS-M, Telephone Interview of Cognitive Status-Modified; pCASL MRI, pseudo Continuous Arterial Spin Labeling MRI; HC BOLD MRI, Hypercapnia Blood Oxygenated Level Dependent MRI. There was no difference between participants' demographic, p > 0.05.

### Neurocognitive measures

Table 2 shows the neurocognitive exam results per the four cognitive domains for physical training and cognitive training groups. Cognitive training group showed improvements in abstraction from complex information as measured by TOSL (Linear gains *p* = 0.01) and working memory as measured by Daneman Carpenter compared to physical training group (Quadratic gains *p* = 0.006). Tests of cognitive performance found that the physical training group significantly improved over training sessions in immediate and delayed text-level memory relative to the cognitive training group. Specifically, two measures of memory function, immediate and delayed text memory recall, showed improvement from T1 to T3, *p* = 0.01 and 0.007, respectively. Additionally, we compared cognitive training participants with MRI scan (*n* = 13) to those without MRI scan (*n* = 5) and found no differences on cognitive measures (*p* > 0.10, Table S1), implying that attrition occurred at random.

**Table 2:** **Neurocognitive measurements (mean ± *S.E.M.*)**.

	**Physical training (PT)**			**Cognitive training (CT)**			***F***		p-values^*^		**η^2^**	
	**T1**	**T2**	**T3**	**T1**	**T2**	**T3**	**Linear**	**Quad**	**Linear**	**Quad**	**Linear**	**Quad**
**COMPLEX ABSTRACTION**												
TOSL (rs)	4.9 ± 0.4	4.6 ± 0.4	4.4 ± 0.4	4.7 ± 0.4	5.6 ± 0.4	5.6 ± 0.4	7.53	0.90	0.01^§^	0.35	0.18	0.03
**EXECUTIVE FUNCTION**												
WAIS-III similarities (ss)	12.3 ± 0.5	12.9 ± 0.5	13.8 ± 0.5	12.9 ± 0.5	13.9 ± 0.5	14.9 ± 0.5	0.83	0.14	0.37	0.71	0.02	0.004
Daneman carpenter (rs)	2.5 ± 0.2	2.5 ± 0.2	2.8 ± 0.2	2.8 ± 0.2	3.3 ± 0.2	3.1 ± 0.2	0.14	8.69	0.71	0.006^§^	0.004	0.21
DKEFS sorting contrast (ss)	10.5 ± 0.5	10.4 ± 0.5	11.3 ± 0.5	10.6 ± 0.5	9.8 ± 0.5	11.0 ± 0.5	0.21	0.38	0.65	0.54	0.01	0.01
COWAT category (ss)	10.5 ± 0.6	11.2 ± 0.6	11.4 ± 0.6	11.8 ± 0.6	12.6 ± 0.6	12.6 ± 0.6	0.05	0.09	0.82	0.77	0.002	0.003
COWAT letter (rs)	38.2 ± 2.3	42.4 ± 1.3	42.9 ± 2.3	43.5 ± 2.3	46.7 ± 2.3	46.7 ± 2.3	0.34	0.01	0.57	0.94	0.01	0.000
Trails B (rs)	60.6 ± 3.6	56.3 ± 3.6	54.9 ± 3.6	58.6 ± 3.6	56.2 ± 3.6	50.9 ± 3.6	0.15	0.52	0.70	0.48	0.004	0.015
**MEMORY**												
CVLT total (rs)	57.4 ± 2.3	59.4 ± 2.4	62.6 ± 2.3	55.9 ± 2.3	55.0 ± 2.3	62.0 ± 2.3	0.07	2.13	0.80	0.16	0.002	0.064
WMS immediate LM (ss)	15.2 ± 0.7	15.7 ± 0.7	16.8 ± 0.7	16.1 ± 0.7	15.7 ± 0.7	14.7 ± 0.7	7.28	0.40	0.01^§^	0.53	0.18	0.01
WMS delayed LM (ss)	13.5 ± 0.8	14.4 ± 0.8	15.5 ± 0.8	14.3 ± 0.8	13.8 ± 0.8	13.1 ± 0.8	8.21	0.02	0.007^§^	0.88	0.199	0.001
**COMPLEX ATTENTION**												
DKEFS color C1 (ss)	10.8 ± 0.4	10.8 ± 0.4	11.5 ± 0.4	11.2 ± 0.4	11.4 ± 0.4	11.9 ± 0.4	0.04	0.26	0.85	0.61	0.001	0.008
DKEFS word reading C2 (ss)	9.6 ± 0.5	9.8 ± 0.5	10.4 ± 0.5	11.4 ± 0.5	11.8 ± 0.5	11.8 ± 0.5	0.30	0.49	0.59	0.49	0.009	0.015
DKEFS inhibition C3 (ss)	11.4 ± 0.5	12.0 ± 0.5	12.0 ± 0.5	12.3 ± 0.5	12.3 ± 0.5	12.6 ± 0.5	0.57	2.26	0.46	0.14	0.017	0.064
DKEFS inhibit/Switch C4 (ss)	12.2 ± 0.4	12.5 ± 0.4	12.9 ± 0.4	12.6 ± 0.4	12.7 ± 0.4	13.3 ± 0.4	0.00	0.15	0.96	0.70	0.000	0.005
WAIS digit span forward	9.9 ± 0.5	10.2 ± 0.5	10.3 ± 0.5	12.0 ± 0.5	11.7 ± 0.5	11.9 ± 0.5	0.58	0.48	0.45	0.49	0.017	0.014
WAIS digit span backward	7.2 ± 0.5	7.6 ± 0.5	7.5 ± 0.5	8.0 ± 0.5	8.9 ± 0.5	8.0 ± 0.5	0.25	2.44	0.62	0.13	0.007	0.067

TOSL, Test of Strategic Learning; WAIS, Wechsler Adult Intelligence Scale; DKEFS, Delis Kaplan Executive Function System; COWAT, Controlled Oral Word Association Test; CVLT, California Verbal Learning Test; WMS, Wechsler Memory Scale; LM, Logical Memory; ss, standard score; rs, raw score.

* p-values refers to specified tests of interaction contrasts.

§ Indicates significance at 10% false discovery rate (FDR) over the set of simultaneous tests.

### Physiological measures

Several physiological parameters were measured at baseline-, mid-, and end-of physical training to assess participants' fitness level, as shown in Table 3. The VO_2_ Max, a gold standard for measuring physical fitness, failed to reach statistical significance when compared to the cognitive training group (Linear: *p* = 0.17 and Quad: *p* = 0.15). This may be in part due to short duration of physical training program of 12 weeks. The RPE, however, decreased (i.e., lower exertion rate) from T1 to T3 in the physical training group compared to cognitive training group linearly, *p* = 0.02.

**Table 3:** **Physiological measurements (mean ± *S.E.M*)**.

**Physiological measures**	**Physical training (PT)**			**Cognitive training (CT)**			***F***		p-values^*^		***η^2^***	
	**T1**	**T2**	**T3**	**T1**	**T2**	**T3**	**Linear**	**Quad**	**Linear**	**Quad**	**Linear**	**Quad**
Body weight (kg)	77.2 ± 3.7	77.7 ± 3.7	77.5 ± 3.7	76.7 ± 3.7	76.9 ± 3.7	76.9 ± 3.7	0.00	0.44	0.99	0.51	0.000	0.013
Max heart rate (bpm)	152.4 ± 3.4	152.6 ± 3.4	152.6 ± 3.4	152.9 ± 3.4	154.9 ± 3.4	149.5 ± 3.4	0.69	1.71	0.41	0.20	0.021	0.049
VO2 Max (ml/kg/min)	19.3 ± 1.0	20.9 ± 1.0	20.4 ± 1.0	20.7 ± 1.0	20.9 ± 1.0	20.6 ± 1.0	2.01	2.18	0.17	0.15	0.057	0.062
EtCO2 (mmHg)	11.7 ± 0.6	11.9 ± 0.7	11.4 ± 0.4	11.7 ± 0.7	12.2 ± 0.7	11.7 ± 0.6	0.17	0.03	0.68	0.87	0.007	0.001
RPE	16.2 ± 0.5	14.9 ± 0.5	15.3 ± 0.5	15.4 ± 0.5	16.6 ± 0.5	16.6 ± 0.5	6.16	3.18	0.02^§^	0.08	0.157	0.088

VO_2_ Max, Maximal Oxygen Consumption, RPE, Rating of Perceived Exertion (RPE), etCO_2_, End Tidal CO_2_ between normocapnia and hypercapnia.

* p-values refers to specified tests of interaction contrasts.

§ Indicates significance at 10% false discovery rate (FDR) over the set of simultaneous tests.

### MRI measurements

**Figure 1:**
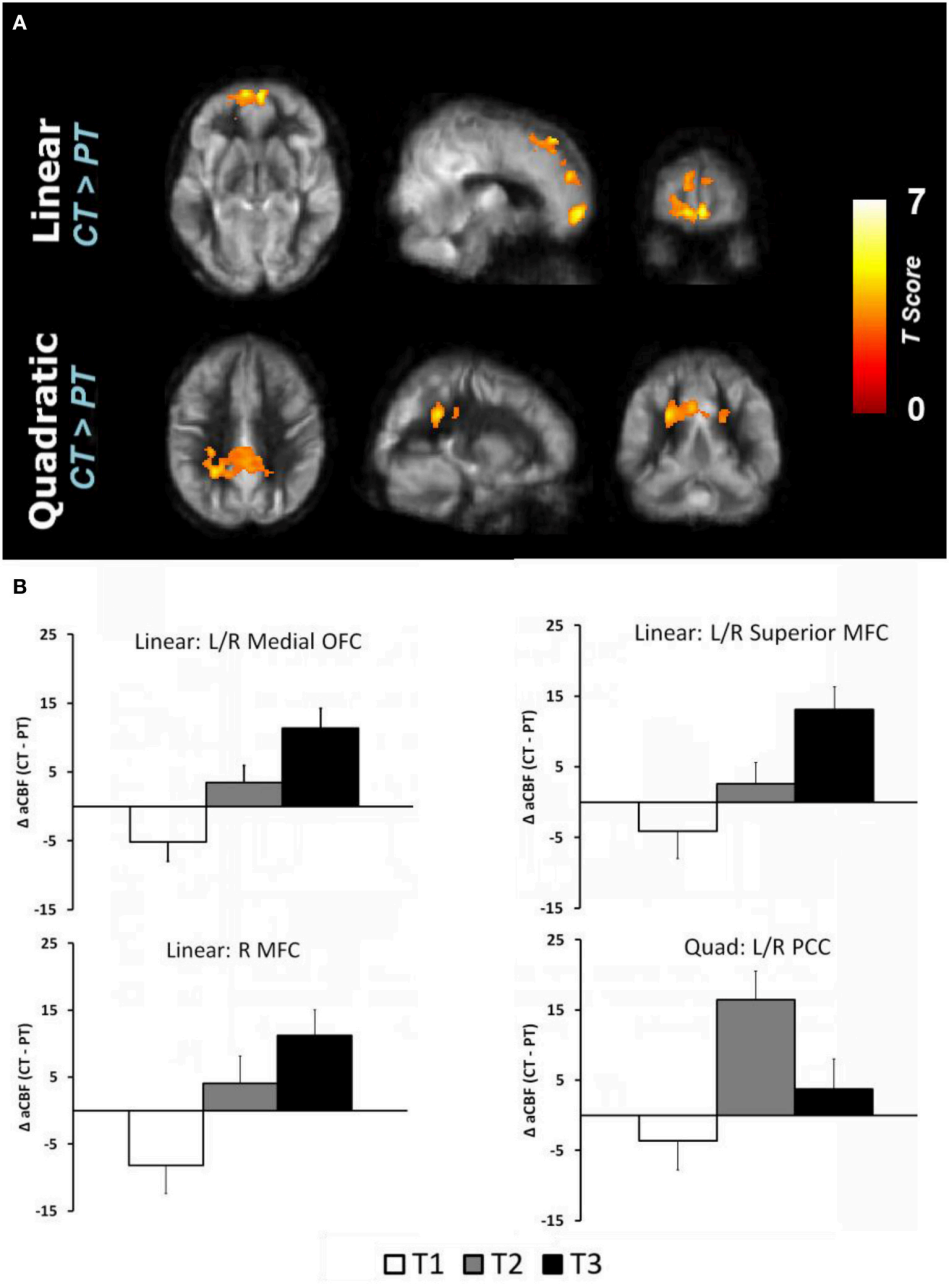
**(A)** Reultss of voxel based analysis are superimposed on an average CBF map of all participants for linear (T1 to T3 change) and quadratic (maximal T2 change) interaction contrasts at *p* < 0.01 (FWE corrected) and *k* ≥ 1232 mm^3^. The regions experiencing a linear increase are located in the frontal regions while the regions experiencing a quadratic pattern of CBF increase are located in the posterior regions. **(B)** The regional CBF difference between CT and PT (i.e., CT–PT) groups are shown to depict linear and quadratic trends. For instance, the CBF of bilateral medial OFC increases from T1 to T2 and T3, i.e., linear trend, whereas the bilateral posterior cingulate cortex (PCC) increases from T1 to T2 and then decreases at T3, i.e., quadratic trend.

The global CBF at T1 for physical training and cognitive training groups were similar at baseline (*p* = 0.94); 46.8 and 47.0 ml/100 g/min, respectively. The cognitive training group's global CBF increased by 7.9% from T1 to T2 and remained elevated (7.9%) at T3 (Linear: *p* = 0.05 and Quadratic: *p* = 0.09) whereas the physical training group's global CBF did not change significantly (Linear: *p* = 0.18 and Quadratic: *p* = 0.58). The global CBF change in cognitive training group was significantly different compared to physical training group (Linear: *p* = 0.025, Quadratic: *p* = 0.18). To evaluate which brain regions may have contributed to the CBF increase, we conducted a voxel-based analysis (VBA). Figure 1A shows the VBA results between cognitive training and physical training groups (i.e., CT > PT), testing whether the CBF differences increase monotonically from T1 to T3 (i.e., Linear, also referred as “at T3”) or whether the CBF changed maximally at T2 (i.e., quadratically, also referred as “at T2”). The cognitive training group showed a significant increase in blood flow at T3 in bilateral medial orbital frontal, bilateral superior medial frontal and right middle frontal cortices compared to the physical training group, shown in Figure 1A. Additionally, the cognitive training group showed a peak increase at T2 (i.e., Quadratic) in the bilateral middle/posterior cingulate cortices compared to the physical training group, also shown in Figure 1A. The reverse contrast (i.e., CT < PT), however, did not yield any significant results. Table 4 summarizes these findings with a FWE maintained at 0.01 and cluster volume ≥ 1232 mm^3^.

**Table 4:** **CBF regions that showed significant blood flow increase at rest in cognitive training compared to physical training group**.

**Brain regions**	**BA**	**Cluster size (mm^3^)**	***MNI***			***T*-values**
			***X***	***Y***	***Z***	
**LINEAR (CT** > **PT)**						
L/R medial orbitofrontal cortex	11/10	11,544	6	64	−8	5.47
L/R superior medial frontal cortex	6/8/32	5768	8	42	56	5.12
R middle frontal cortex	4/6	7352	30	26	32	4.52
**QUADRATIC (CT** > **PT)**						
L/R Middle/Posterior cingulate cortex	23/31	11,144	−24	−48	32	4.56

L/R, Left/Right; BA, Brodmann Area; MNI, Montreal Neurological Institute; CT, Cognitive Training; PT, Physical Training.

The regional CBF-values of the aforementioned regions were extracted and plotted in Figure 1B to show linear and quadratic trends. For instance, the CBF of the bilateral medial orbitofrontal cortex increases monotonically from T1 to T2 and T3. Applying these clusters to the CVR maps, however, did not show any change across the groups (*p* > 0.19). The lack of CVR difference between groups implies an increase in neuronal demand rather than vasculature change in the CT group.

**Figure 2:**
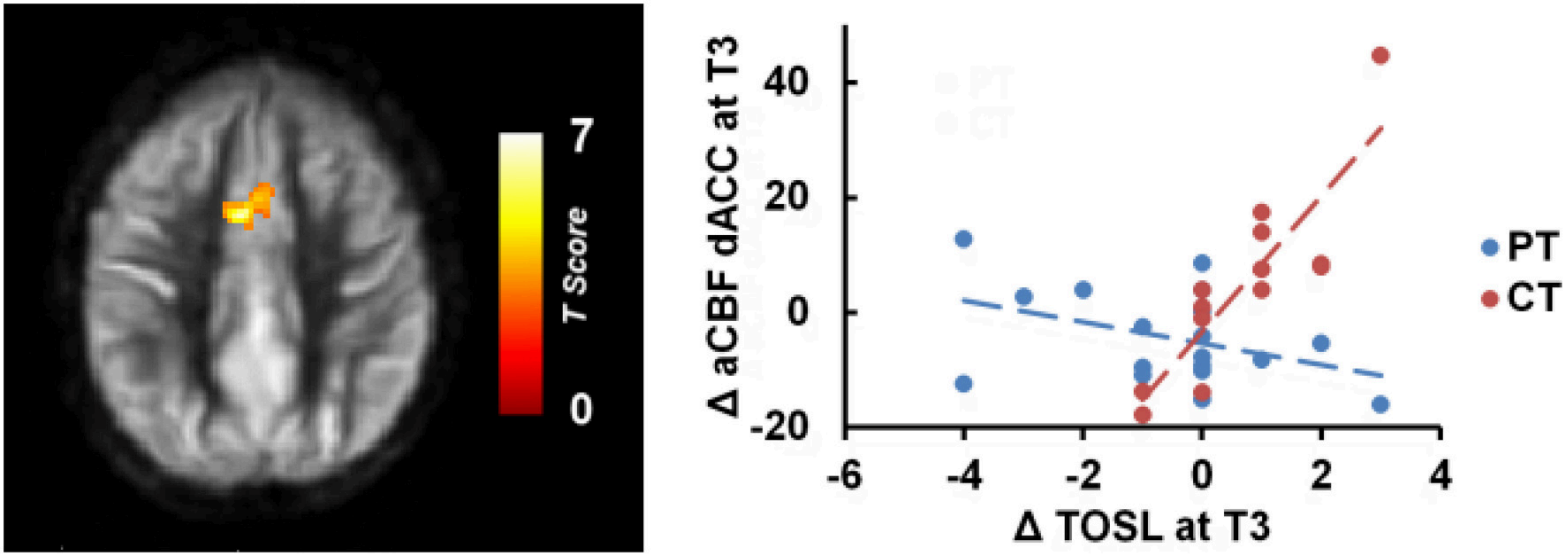
**CT group showed significant association between gains in regional CBF and behavioral measures compared to PT group**. The CT group's TOSL (strategic reasoning) score at T3 also showed significant association to bilateral dACC CBF increase at T3 compared to PT group, *p* < 0.05 [FWE Corrected]. “at T3” refers to linear trend (i.e., change from T1 to T3).

Whole brain voxel-wise correlation analysis between complex abstraction (i.e., TOSL) score and CBF maps (Linear: T3–T1) showed significant correlation to bilateral dorsal anterior cingulate cortex (dACC), BA 32, CBF in CT group compared to PT group; MNI coordinates: [−4 +10 +38], t-score of 6.35 and cluster size = 3232 mm^3^ (*p* < 0.01 [FWE Corrected] and cluster size ≥ 752 mm^3^), shown in Figure 2. Additionally, no relationship between complex abstraction (i.e., TOSL)/CVR, CBF/Daneman Carpenter or CVR/Daneman Carpenter was found at (*p* < 0.01 [FWE Corrected] and cluster size ≥ 752 mm^3^).

**Figure 3:**
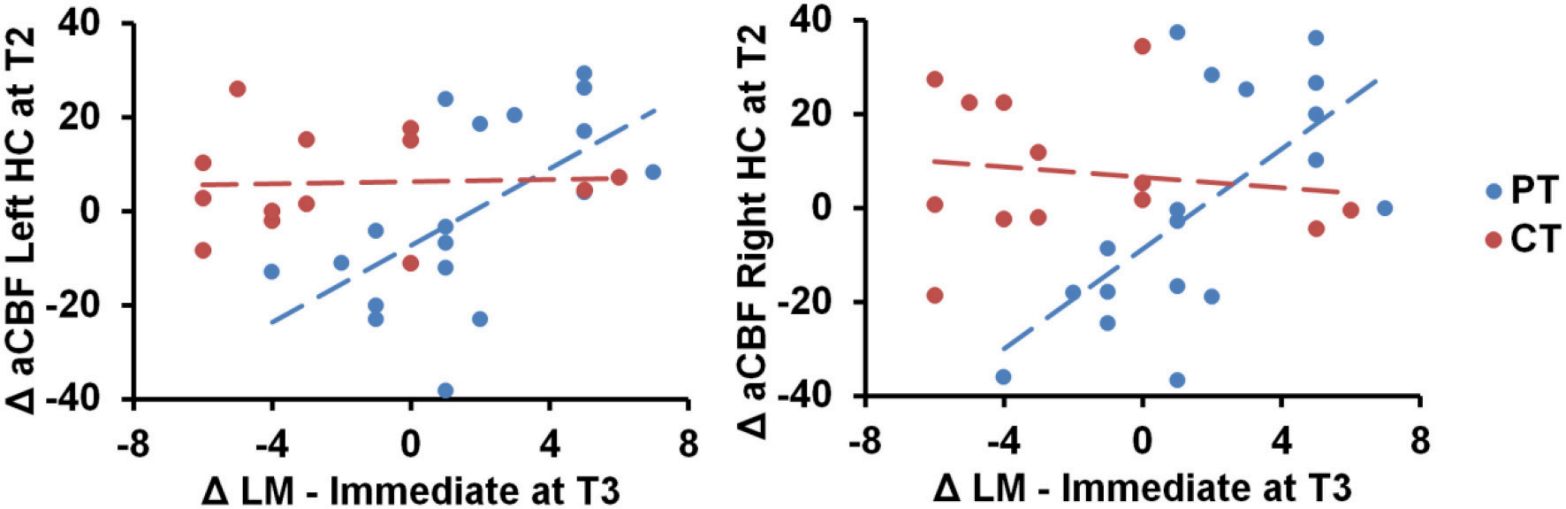
**Scatterplots of immediate logical memory (LM) against the left/right hippocampus (HC) CBF regions are shown**. The PT group showed positive immediate logical memory (LM) change at T3 against maximal T2 change of CBF in the left and right hippocampus, *p* = 0.01 and 0.003, respectively. “at T3” refers to linear trend (i.e., change from T1 to T3) and “at T2” refers to quadratic trend (i.e., maximal T2 change).

We also conducted ROI analyses of hippocampi CBF. The absolute CBF of hippocampus did not change significantly between cognitive training and physical training groups (Left hippocampus—Linear: *p* = 0.11 and Quad: *p* = 0.29, right hippocampus—Linear: *p* = 0.18 and Quad: *p* = 0.34). However, the physical training group's immediate logical memory showed a significant association to the left/right hippocampus CBF changes compared to the cognitive training group, *p* = 0.01 and 0.003, respectively, as shown in Figure 3. We additionally conducted Pearson correlation for just the PT group between left/right hippocampus and immediate logical memory. We found that the PT group had a significant correlation between both left hippocampus and immediate logical memory (*r* = 0.60, *p* = 0.008) as well as right hippocampus and immediate logical memory (*r* = 0.64 and *p* = 0.004). On the other hand, no significant relationship was found between immediate logical memory and left/right hippocampus CVR, *p* = 0.13 and 0.53, respectively. Voxel-wise covariate analysis of RPE measures against CBF data did not yield any significant clusters at *p* [FWE Corrected] < 0.01 and cluster size ≥ 752 mm^3^.

## Discussion

Cognitive and aerobic exercise trainings have emerged as two promising non-pharmacological interventions to enhance cognitive brain health in normally aging adults. Most studies have focused on either a single training protocol (Jaeggi et al., 2008; Takeuchi et al., 2013) or combined cognitive and physical training to alter the course of cognitive decline in healthy aging (Jak, 2011). In this study, however, we aimed to elucidate the distinct impact of each training on the brain and behavior. We found that the cognitive training improved executive function whereas physical training enhanced memory. Furthermore, cognitive training group showed CBF increase in the prefrontal and posterior cingulate cortices compared to physical training group. Interestingly, we found that the gain in near transfer effect (i.e., TOSL) associated with increase in dACC CBF in the CT group and the enhanced memory correlated with increase in hippocampal CBF in the PT group. Lastly, evidence of increased resting blood flow without changes to vascular reactivity implicates increased neural health rather than improved vascular response. These preliminary results support a realizable potential to improve brain health through short-term cognitive and aerobic training protocols.

First, the present results conferred that distinct benefits can be realized from either the cognitive training (SMART) or aerobic exercise training in normally aging middle to older age adults. We identified improvement in complex abstraction and working memory in the cognitive training group as well as enhanced immediate and delayed memory in the physical training group. Cognitive trainings that engage and challenge “integral/complex function” have previously shown to engage frontally-mediated control processes and achieve broader impact on associated cognitive domains and daily function than those achieved through training specific processes (D'Esposito et al., 2006; Badre and D'Esposito, 2009; Anguera et al., 2013). Prior clinical trials examining the specific effects of the cognitive training (SMART) protocol in adult populations revealed similar performance gains across a wide array of executive function measures including abstraction of complex information/goal setting, switching, concept abstraction, working memory, fluency, and non-verbal reasoning (Anand et al., 2011; Chapman and Mudar, 2014; Chapman et al., 2015; Vas et al., 2015). Whereas, improvement in complex abstraction may be viewed as limited to a near transfer effect for our CT group; the importance of this gain is supported by extant evidence that this is a ubiquitous and foundational top-down cognitive skill called upon in everyday life (Zwaan and Radvansky, 1998; Chapman and Mudar, 2014). Additionally, a far transfer gain was also identified in working memory. Training individuals to actively construct abstracted meanings from the incoming stimuli reinforces the human brain's capacity to better understand and remember generalized meanings than detailed ideas (Gabrieli, 2004). With regard to aerobic training benefits, we demonstrated significant increases in both immediate and delayed memory. Enhanced memory performance has been a commonly identified benefit associated with physical activity (Erickson et al., 2011). The health significance of the positive memory gains is underscored by extensive evidence that memory loss is the most frequent complaint and concern in aging (Jonker et al., 2000). Our results support the potential for the adopted cognitive training (SMART) protocol to improve executive function capacities and for aerobic exercise to increase memory performance in healthy older adults ages 56 to 75.

Secondly, we found different regions and patterns of change in resting CBF between the two training protocols. The physical training group showed positive relationship between memory and CBF changes in the exercise group; specifically, gains in immediate logical memory were associated with increased resting CBF in bilateral hippocampal regions. The cognitive training group, on the other hand, showed significant blood flow increase in key brain region: prefrontal, PCC, and dACC. The regions with CBF increases are identified in both task positive [fronto-parietal (FP) and cingulo-opercular (CO)] as well as task negative networks [default mode network (DM); Dosenbach et al., 2007]. The FP network operates at fast time-scales for moment-to-moment adaptive task control whereas the CO network operates over longer time-scales for stable task maintenance (Dosenbach et al., 2008). On the other hand, PCC is a key region of default-mode network and is known to be vulnerable to dementia and aging (Raz et al., 1997; Kennedy and Raz, 2009). The linear increase in CBF for prefrontal cortical regions, associated with top-down cognitive control and abstraction, suggests the potential benefit of the cognitive training did not plateau within the time period of our study, with a concomitant linear improvement in complex abstraction (see Table 2). The quadratic increase (i.e., maximal change at T2) in CBF in the posterior cingulate may be due to rapid changes associated early in the cognitive training regimen which then plateaued, perhaps because neuronal changes were consolidated.

We propose that the distinct benefits of CT resulted from harnessing experienced-driven neural plasticity; whereby the cognitive strategies engaged and boosted neural activity and consequently CBF in nodes across functionally-related cognitive control or top-down processing networks. Exercise-induced benefits, on the other hand, may have enhanced memory capacity through enhancement of the inherent metabolic activity in more focal brain regions, i.e., the hippocampi (Burdette et al., 2010). Prior work has shown that cardiovascular activity increases hippocampal blood flow and volume (Niemann et al., 2014). Our pattern of higher CBF in the hippocampi being linked to improved immediate memory for the physical training group is consistent with prior work showing that longer term exercise of 1-year increased the size of anterior hippocampal volume, improved memory function and increased serum levels of BDNF, which mediates neurogenesis (Erickson et al., 2011). Increases in CBF associated with exercise-induced memory gains may potentially represent a surrogate marker for neurogenesis (Pereira et al., 2007).

Prior work has shown resting state CBF to be a promising neural marker of brain change from distinct interventions (Mozolic et al., 2010; Vas et al., 2015). Controversy exists as to whether increased CBF reflects positive or negative changes in brain function (Sojkova et al., 2008). We suggest that the present evidence supports positive brain changes since the increased resting CBF changes were associated with improved cognitive performance in both CT and PT groups. Specifically, we found that higher performance in complex abstraction was linked to increase in resting CBF in the dACC in the CT group and in resting CBF in hippocampi for those with improved memory in the PT group. We cautiously interpret our findings and recognize that continued improvements over longer intervals may be associated with decreased resting CBF. Additionally, there may be a different pattern of associations between CBF and cognitive gains in activation studies when compared to resting-state brain studies.

The present findings provide some of the first evidence identifying the distinct neural and cognitive benefits of cognitive challenges vs. aerobic exercise in healthy older adults. We argue that cognitive training as reflected through the SMART protocol could potentially contribute to rebuilding neural health in a declining neural system where age-related brain losses have been previously identified. Global and regional CBF, particularly in the frontal networks, have been shown to be vulnerable to the process of senescence (Lu et al., 2011). The potential to enhance neural health (i.e., increase neuronal activity) with cognitive reasoning training is implicated by the lack of significant changes in cerebrovascular reactivity. A tight coupling between brain activity during active mental processes being mirrored to a large degree in intrinsic resting brain has been developed by a number of neuroscientists (Raichle, 2015). Combining this new finding of increased CBF with no concomitant change in CVR with results from our prior study comparing the cognitive reasoning to a wait list control adds further to the view that neural health was enhanced by the advanced reasoning training in normally aging adults (Chapman et al., 2015).

The benefits from short term aerobic exercise training were manifested through lower RPE, higher immediate/delayed memory scores, and increased resting CBF in bilateral hippocampi regions for those with improved immediate memory scores. We interpreted this finding to suggest that brain changes with short-term aerobic exercise are selective rather than global, similar to what has been suggested by other researchers (Burdette et al., 2010). Whereas, there was not a statistically significant increase in VO_2_ Max in the physical training group, there was a small albeit non-significant increase in VO_2_ Max. We believe the failure to find significant differences may have been due to the short duration of intervention (i.e., 12 weeks) and the somewhat cautious exercise prescription given their prior sedentary lifestyle. Alternatively in a previous arm of this larger project, we did find that the aerobic exercise group significantly improved physical fitness when compared to the wait-list control group (Chapman et al., 2013). We postulate that perhaps the cognitive training group in the present study increased their exercise regimen since their fitness was being evaluated at T2 and T3. Future studies could monitor the possibility of increased exercise across training groups by employing wearable fitness monitoring technology. Additionally, the finding of a lower RPE following their brief training regimen is intriguing. We interpret these findings to suggest that the decrease in RPE may be related to the conditioning from regular exercise leading to lower perceived exertion or that they were acclimated to the exertion by the prior one or two treadmills and hence, reported lower RPEs. The role of perceived effort may be a factor in the degree to which individuals continue with a sustained exercise effort.

We failed to find increased cardiovascular fitness in this 3-month exercise training trial. This is consistent with most prior literature that has failed to find that higher cardiorespiratory fitness is linked to improved cognition (Young et al., 2015). The relationships across cardiovascular fitness, neural health, and cognitive function are equivocal and require continued examination. For example, a recent study failed to find a relationship between exercise-induced increases in white matter integrity and improved memory performance, despite reporting a link between cardiovascular fitness and white matter integrity in a 1-year exercise trial (Voss et al., 2013). In our study, we found a significant association between improved memory and increases in CBF but not in changes to cardiovascular fitness. Whereas, prior work has suggested that aerobic exercise improves executive control functions, the benefits were not apparent in this cognitive domain in our study (Colcombe et al., 2004), confirming similar findings in a longer-term exercise trial (Voss et al., 2013).

## Conclusion and limitations

This study has a number of strengths which include the following: a randomized group-design, inclusion of both brain and neurocognitive outcome measures, utilization of two functional MRI techniques to assess CBF and cerebrovascular functions, documented benefits with relatively short-term interventions, and promising findings on non-pharmacological interventions that could be practiced independently, making them practical and affordable. We also note a number of limitations of the study including: a small sample size, short-duration of physical training to show brain/physiological benefits, lack of long-term follow-up assessments beyond 12 weeks, limited age range (56–75; failure to generalize to those 55 and younger or those older than 75) and failure to obtain prior experience of participants' cognitive and physical training as well as limited control between and within group training for participants' social interactions, physical exercise, and cognitive practice outside of training. Thus, we interpret our findings cautiously; however, we do not believe that these limitations diminish the potential implications nor weaken the findings of distinct brain and cognitive differences between advanced reasoning training and aerobic exercise. One might be concerned that differences in the social aspect of the trainings could explain the group differences in brain or cognitive measures. We do not feel this is the case for two reasons. First, it is difficult to judge which group had the most social attention since the CT group met for 12 sessions only in group settings and the PT group had opportunity to interact with the trainer in 36 training sessions. Secondly in a cognitive training trial in aging adults with well-controlled social contact, socialization was not found to have a significant impact on cognitive function (Park et al., 2014). We do not feel that the younger cohort of this study is a limitation. There is little consensus as to what is the best age to intervene to address age-related losses. We were interested in studying a somewhat younger cohort than prior studies starting with middle to older age adults (55+) since this is the age when cognitive decline reportedly accelerates (Agarwal et al., 2009). According to the recent IOM Report (2015), there is growing consensus that greater benefit may be derived by starting younger instead of waiting to intervene at later ages when major cognitive and brain losses have already accrued.

The present results provide promising clinically-relevant evidence that aerobic exercise and cognitive reasoning training contribute differentially to advancing brain health. With regard to cognitive training, we propose that enhancing executive functions and the supporting neural systems through advanced reasoning training could increase the resilience of neural function and overall cognitive brain health, based on work by Dosenbach et al. (2008). In the first known training trial to deploy both CBF and CVR, we found novel evidence that the brain benefits from cognitive training were due to increased neuronal demand rather than vascular change. This finding is consistent with Shatil et al.'s proposed interpretation that cognitive training may be the main agent for cognitive gains in older adults when compared to the benefit of exercise (Shatil, 2013). We found that exercisers with greater memory gains showed higher CBF in hippocampal regions, an area particularly vulnerable to aging and dementia.

At a global level, this study highlights the distinct brain and cognitive benefits that may be harnessed by incorporating healthy habits, such as complex reasoning and aerobic exercise, into one's life style. Future trials are needed to determine whether additive effects are realizable by combined protocols, such as reasoning training/physical exercise, and if these practices serve as potential neuroprotective agents to promote successful cognitive aging.

## Author contributions

SC: designed the study, interpreted the results, and wrote the manuscript; SA: performed neuroimaging analysis, interpreted the results, and wrote the manuscript; JS: assisted with statistical design and revised the manuscript; MK: performed cognitive training program and scored the behavioral data; LD: supervised the physical training program; ND: performed neuropsychological exams and statistical analysis; AP: performed literature review; HL: designed the neuroimaging protocol, interpreted the results, and revised the manuscript; MD: interpreted the results and revised the manuscript.

### Conflict of interest statement

The authors declare that the research was conducted in the absence of any commercial or financial relationships that could be construed as a potential conflict of interest.
