# Supplementary material for: Distinct Brain and Behavioral Benefits from Cognitive vs. Physical Training: A Randomized Trial in Aging Adults
Source: Front Hum Neurosci. 2016 Jul 18;10:338. doi: 10.3389/fnhum.2016.00338 (PMC4939293; doi:10.3389/fnhum.2016.00338)
Supplement: Supplementary file 1 [file DataSheet1.docx]

**Figure S1.** Flow diagram of the progress through the phases of a parallel randomized trial of 3 groups.

**Table S1.** Neuropsychological exam results for the Cognitive Training group with and without MRI scans (mean ± S.E.M.)

** HC Issues – Hypercapnia (HC) machine malfunctioned (e.g. no capnography recording), participant’s nose clip fell off, subject hyper- or hypo-ventilated during the experiment.*

**Analyzed**

**Cognitive Exam (n=18)**

**Excluded (n=0)**

**Physical Exam (n=18)**

**Excluded (n=0)**

**CBF (n=13)**

**Excluded (n=5)**

**High motion (n=1)**

**Artefact (n=1)**

**No MRI (n=3)**

**CVR (n=12)**

**Excluded (n=6)**

**HC issues (n=1)**

**High motion (n=1)**

**Declined HC (n=1)**

**No MRI (n=3)**

**Analysis**

**Allocation**

**Mid-Point**

**Follow-Up**

**Analyzed**

**Cognitive Exam (n=19)**

**Excluded (n=0)**

**Physical Exam (n=19)**

**Excluded (n=0)**

**CBF (n=18)**

**Excluded (n=1)**

**High motion (n=0)**

**Artefact (n=0)**

**No MRI (n=1)**

**CVR (n=12)**

**Excluded (n=7)**

**HC issues (n=4)**

**High motion (n=1)**

**Declined HC (n=1)**

**No MRI (n=1)**

**Analyzed**

**Cognitive Exam (n=18)**

**Excluded (n=0)**

**Physical Exam (n=18)**

**Excluded (n=0)**

**CBF (n=18)**

**Excluded (n=0)**

**High motion (n=0)**

**Artefact (n=0)**

**No MRI (n=0)**

**CVR (n=14)**

**Excluded (n=4)**

**HC issues (n=3)**

**High motion (n=0)**

**Declined HC (n=1)**

**No MRI (n=0)**

**Cognitive Training (n=25)**

**Control (n=21)**

**Physical Training (n=21)**

**Lost to mid-point (n=3)**

**Lost to mid-point (n=2)**

**Lost to mid-point (n=7)**

**Lost to follow-up (n=0)**

**Lost to follow-up (n=0)**

**Lost to follow-up (n=0)**

**Enrollment**

**Assessed for eligibility**

**(n=137)**

**Excluded (n=70)**

**Not meeting inclusion criteria (n=70)**

**Randomized (n=67)**

|  | **CT without MRI** (n=5) | | | **CT with MRI** (n=13) | | | ***p-value*** | |
| --- | --- | --- | --- | --- | --- | --- | --- | --- |
|  | **T1** | **T2** | **T3** | **T1** | **T2** | **T3** | **Linear** | **Quad** |
| ***Complex Abstraction*** |  |  |  |  |  |  |  |  |
| TOSL (rs) | 4.4±0.8 | 5.4±0.8 | 6.0±0.5 | 4.8±0.5 | 5.7±0.5 | 5.5±0.3 | 0.24 | 0.73 |
|  |  |  |  |  |  |  |  |  |
| ***Executive Function*** |  |  |  |  |  |  |  |  |
| WAIS-III Similarities (ss) | 12.4±1.0 | 12.8±0.9 | 13.6±0.8 | 13.1±0.7 | 14.3±0.5 | 15.4±0.5 | 0.26 | 0.64 |
| Daneman Carpenter (rs) | 2.5±0.3 | 2.8±0.3 | 2.8±0.4 | 2.9±0.2 | 3.5±0.2 | 3.3±0.2 | 0.84 | 0.30 |
| DKEFS Sorting Contrast (ss) | 11.0±1.5 | 9.4±0.9 | 10.6±1.0 | 10.5±0.9 | 10.0±0.6 | 11.2±0.6 | 0.51 | 0.65 |
| COWAT Category (ss) | 10.6±1.1 | 12.2±0.8 | 12.0±0.8 | 12.2±0.7 | 12.8±0.5 | 12.8±0.5 | 0.40 | 0.35 |
| COWAT Letter (rs) | 39.0±4.4 | 45.2±3.3 | 43.8±3.6 | 45.2±2.7 | 47.3±2.0 | 47.8±2.3 | 0.53 | 0.29 |
| Trails B (rs) | 59.6±6.2 | 56.2±6.9 | 57.0±6.4 | 58.2±3.8 | 56.2±4.3 | 48.6±3.9 | 0.43 | 0.51 |
|  |  |  |  |  |  |  |  |  |
| ***Memory*** |  |  |  |  |  |  |  |  |
| CVLT Total (rs) | 55.2±4.3 | 55.4±4.6 | 60.6±4.3 | 56.2±2.7 | 54.9±2.8 | 62.5±2.6 | 0.87 | 0.58 |
| WMS Immediate LM (ss) | 15.0±1.4 | 15.6±1.1 | 15.2±1.6 | 16.5±0.9 | 15.8±0.7 | 14.5±1.0 | 0.29 | 0.92 |
| WMS Delayed LM (ss) | 13.6±1.1 | 15.0±1.4 | 13.2±1.9 | 14.5±0.7 | 13.3±0.9 | 13.1±1.2 | 0.61 | 0.19 |
|  |  |  |  |  |  |  |  |  |
| ***Complex Attention*** |  |  |  |  |  |  |  |  |
| DKEFS Color C1 (ss) | 10.8±0.6 | 10.4±0.9 | 12.2±0.8 | 11.4±0.4 | 11.8±0.6 | 11.8±0.5 | 0.22 | 0.10 |
| DKEFS Word Reading C2 (ss) | 11.6±0.8 | 11.8±0.7 | 12.8±0.7 | 11.3±0.5 | 11.8±0.4 | 11.5±0.5 | 0.14 | 0.15 |
| DKEFS Inhibition C3 (ss) | 13.0±1.1 | 12.8±1.0 | 12.8±0.8 | 12.1±0.7 | 12.1±0.6 | 12.5±0.5 | 0.44 | 0.93 |
| DKEFS Inhibit/Switch C4 (ss) | 12.8±0.8 | 13.6±1.1 | 13.6±0.6 | 12.5±0.5 | 12.3±0.7 | 13.2±0.4 | 0.76 | 0.31 |
| WAIS Digit Span Forward | 11.6±0.9 | 11.0±0.9 | 11.4±0.7 | 12.2±0.6 | 11.9±0.5 | 12.1±0.4 | 0.90 | 0.76 |
| WAIS Digit Span Backward | 8.4±1.0 | 8.8±0.9 | 8.0±1.0 | 7.8±0.6 | 9.0±0.5 | 8.0±0.6 | 0.68 | 0.60 |

*TOSL-Test of Strategic Learning; WAIS- Wechsler Adult Intelligence Scale; DKEFS-Delis Kaplan Executive Function System; COWAT-Controlled Oral Word Association Test; CVLT-California Verbal Learning Test; WMS-Wechsler Memory Scale; LM-Logical Memory; ss - standard score; rs - raw score.*

**p-value refers to specified tests of interaction contrasts*.

*§ Indicates significance at 10% false discovery rate (FDR) over the set of simultaneous tests*

There was no change (i.e. linear or quadratic) in neuropsychological test scores between Cognitive Training participants who completed MRI and those without MRI scans (p>0.10), implying that attrition was done at random.
